# Increased Synaptophysin Is Involved in Inflammation-Induced Heat Hyperalgesia Mediated by Cyclin-Dependent Kinase 5 in Rats

**DOI:** 10.1371/journal.pone.0046666

**Published:** 2012-10-02

**Authors:** Hong-Hai Zhang, Xiao-Qin Zhang, Wen-Yuan Wang, Qing-Sheng Xue, Han Lu, Jin-Lu Huang, Ting Gui, Bu-Wei Yu

**Affiliations:** 1 Department of Anesthesiology Ruijin Hospital, Shanghai JiaoTong University School of Medicine, Shanghai, P.R. China; 2 Department of Pharmacy, the Sixth Affiliated Hospital of Shanghai JiaoTong University School of Medicine, Shanghai, P.R. China; 3 Department of Anatomy, Institutes of Medical Sciences, Shanghai JiaoTong University School of Medicine, Shanghai, P.R. China; McGill University Department of Neurology and Neurosurgery, Canada

## Abstract

Mechanisms associated with cyclin-dependent kinase 5 (Cdk5)-mediated heat hyperalgesia induced by inflammation remain undefined. This study was designed to examine whether Cdk5 mediates heat hyperalgesia resulting from peripheral injection of complete Freund's adjuvant (CFA) in the spinal dorsal horns of rats by interacting with synaptophysin, a well known membrane protein mediating the endocytosis-exocytosis cycle of synaptic vesicles as a molecular marker associated with presynaptic vesicle membranes. The role of Cdk5 in mediating synaptophysin was examined through the combined use of behavioral approaches, imaging studies, and immunoprecipitation following CFA-induced inflammatory pain. Results showed that Cdk5 colocalized with both synaptophysin and soluble N-ethylmaleimide-sensitive factor (NSF) attachment protein receptors (SNAREs) consisting of VAMP-2, SNAP-25, and syntaxin 1A in spinal dorsal horn of rats. Increased synaptophysin expression of spinal cord horn neurons post intraplantar injection of CFA coincided with increased duration of heat hyperalgesia lasting from 6 h to 3 d. Intrathecal administration of roscovitine, a Cdk5 specific inhibitor, significantly depressed synaptophysin expression during peak heat hyperalgesia and heat hyperalgesia induced by peripheral injection of CFA. Data presented in this report indicated that calpain activity was transiently upregulated 6 h post CFA-treatment despite previous reports suggesting that calpain was capable of cleaving p35 into p25. Results from previous studies obtained by other laboratories demonstrated that significant changes in p35 expression levels within spinal cord horn neurons were not observed in the CFA-treated inflammatory pain model although significant upregulation of Cdk5 kinase was observed between 2 h to 7 d. Therefore, generation of p25 occurred in a calpain-independent fashion in a CFA-treated inflammatory pain model. Our results demonstrated that increased synaptophysin levels were involved in heat hyperalgesia mediated by Cdk5 in spinal cord dorsal horns of CFA-treated rats, suggesting that inhibiting abnormal activation of Cdk5-synaptophysin may present a novel target for diminishing inflammatory pain.

## Introduction

Accumulating evidence has shown that multiple protein kinases are involved in mediating inflammatory pain in the central nervous system (CNS) [1], [2], [3]. It is well known that cyclin-dependent kinase 5 (Cdk5) belongs to a member of the Cdk family whose activities are upregulated during the cell cycle by cyclins [4], [5]. However, Cdk5 activity does not appear to depend on association with cyclins and its activity seems to be confined to postmitotic neurons where it can be activated by p35 or p39 that are cleaved to p25 or p29, respectively, by calpain [6]. Previous studies showed that Cdk5 was mainly associated with mediating neuronal migration and apoptosis [6], [7]. Emerging evidence has shown that Cdk5 plays an important role in mediating heat but not mechanical hyperalgesia associated with inflammatory pain in the dorsal root ganglion (DRG) and in spinal cords of both mice and rats [8], [9], [10], [11]. In the DRG of mice challenged with capsaicin, Cdk5 mediated heat hyperalgesia by phosphorylating the vanilloid receptor 1 (VR1) [8], [9], [10]. In the spinal cords of rats challenged with CFA, studies by others showed that Cdk5 activity and cofactor p25 expression were significantly upregulated and that roscovitine, a selective inhibitor of Cdk family members in neurons, markedly inhibited Cdk5 activity as well as heat hyperalgesia elicited following intraplantar injection of CFA [11]. However, the mechanism of Cdk5-mediated inflammatory pain has remained undefined to date.

Evidence from previous studies indicated that presynaptic Cdk5 was the primary regulator of neurotransmitter release by phosphorylation and downregulation of P/Q-type voltage-dependent calcium channel activity in the central nervous system (CNS) [12], [13]. Furthermore, multiple synaptic vesicle proteins are involved in transporting and releasing neurotransmitters [14], [15]. Synaptophysin is a presynaptic marker representing the first integral synaptic vesicle membrane protein to be cloned [16]. Synaptophysin has been shown to play a primary role in mediating the exocytosis-endocytosis of synaptic vesicles and in regulating the assembly of the soluble N-ethylmaleimide sensitive factor (NSF) attachment protein receptors (SNAREs) complex formation [17], [18]. The SNARE complex is composed of syntaxin 1 A belonging to plasma membrane proteins, the 25 kDa synaptosomal-associated protein (SNAP-25), and the vesicle-associated membrane protein-2 (VAMP-2)/synaptobrevinII [19]. SNARE formation is essential for fusion of vesicles to the plasma membrane [20]. Furthermore, previous studies revealed that overexpression of synaptophysin resulted in increased neurotransmitter release (*e. g*, glutamate release was increased in neuromuscular synapses of xenopus) [21]. In addition, the significant decreases in synaptophysin cluster concentrations caused by Cdk5 conditional knockouts was observed in mouse forebrains [22].

In the brain, synaptophysin is widely distributed and likely to play a role in synaptic transmission [23]. In the spinal cord, synaptophysin expression levels were consistent with the duration of heat hyperalgesia resulting from chronic constriction injury (CCI) of the sciatic nerve in rats demonstrating that synaptophysin modulated heat hyperalgesia was induced by chronic constriction injury [24]. In a sciatic nerve transection model, synaptophysin expression levels were significantly increased in the spinal cord horn after nerve transection [25]. Additionally, previous studies showed that VR1 mediated inflammatory pain by interacting with SNARE proteins [26]. Based on the above evidence, we hypothesized that Cdk5 may mediate heat hyperalgesia induced by inflammation by acting on presynaptic synaptophysin in the spinal cord dorsal horn neurons of rats.

## Materials and Methods

### Animals

Male Sprague-Dawley rats (200∼250g) were used in all experiments. All procedures were approved by the Committee of Animal Use for Research and Education of Shanghai Jiao Tong University School of Medicine. To minimize the number of animals used experiments followed the guidelines established by the Ethical Issues of the International Association for the study of pain [27]. Rats were housed in a controlled environment with 12 h light/dark cycles and access to food and water *ad libitum*. CFA (100 µl, Sigma, St. Louis, MO) was injected into the plantar surface of the ipsilateral hind paw of rats and saline (100 µl) was injected into plantar surfaces of contralateral hind paws as a control (n = 6/group). The ipsilateral and contralateral paws of rats in the control group were injected with same volume of saline as control animals (n = 6/group).

### Surgery and Drug Administration

Drugs were delivered via intrathecal injection as previously described with a slight modification [28]. Briefly, rats were anesthetized with 4% pentobarbital (40 mg/kg) and a catheter (PE-10) was inserted at the lumbar level of the spinal cord between lumbar vertebrates 4 and 5 (L4 and L5). Following recovery from anesthesia and surgery, verification of the proper placement of the catheter was confirmed by injecting 5 µl of 2% lidocaine flushed with 15 µl of saline. Following this procedure, successful insertion of the catheter was confirmed if the presented with impairment of motor function of their hind legs 10 s post lidocaine administration. After surgery (for 5 days) 5 µl of roscovitine (100 µg) (dissolved in 30% DMSO, Sigma) was administered followed by injection of 15 µl sterile saline 30 min before CFA treatment (n = 6/per group). Unless otherwise specified, roscovitine was administered at a concentration of 100 µg in all experiments. The same concentration and volume of DMSO was delivered in the same fashion and served as the vehicle control (n = 6/per group).

### Radiant Heat Paw Withdrawal Test (PWT)

Thermal escape latencies were evaluated using a radiant heat stimulation apparatus as previously described [29]. Briefly, individual rats were placed into a plastic chamber with a 1 mm thick glass that was heated to 30°C by a focused projection bulb located below the glass surface. To avoid burning the skin of rats, the exposure cut-off limit was to 20 s. The escape time between onset of the stimulus and the manifestation of the paw withdrawal response was recorded as the thermal nociceptive latency period. Prior to the behavior test, rats were allowed to acclimate to the chamber for 30 min, and then the thermal stimulus was delivered to the ipsilateral and contralateral hindpaws of rats in all groups. Paw withdrawal latencies (PWLs) was assessed at 0, 6 h, 1 d, 3 d and 5 d post exposure and expressed as an average of 3 trials for each hind paw. The PWL measurement interval between ipsilateral and contralateral paws was 5 min. PWL measurements of rats in the DMSO and roscovitine pretreatment groups was performed in similar fashion.

### Immunofluorescence

Rats were anesthetized with sodium pentobarbital (>100 mg/kg.i.p) and then perfused via the ascending aorta with 200 ml 0.9% saline followed by 4% paraformaldehyde. After perfusion, L4/L5 ipsilateral spinal cord spinal cord dorsal horn tissues were immediately removed and fixed in 4% fixative overnight at 4°C and dehydrated by immersion in 20% sucrose, overnight. The tissues were embedded with Tissue-Tek (Sakura Finetek, Torrance, CA) and frozen in dry ice powder. Transverse sections were cut into 16 µm thick sections at −28°C in a cryostat. For double immunofluorescence, sections were incubated with appropriate antibodies (anti-Cdk5, 1∶200, Abcam [Cambridge, MA] ab115812 and ab40773; anti-synaptophysin, 1∶200, Abcam [Cambridge, MA] ab8049; anti-VAMP-2, 1∶200, Abcam [Cambridge, MA] ab3347; anti-syntaxin 1A, 1∶100, Abcam [Cambridge, MA] ab41453; anti-snap-25, 1∶100, Abcam [Cambridge, MA] ab53723; anti-p-35, 1∶100, Cell Signaling [Danvers, MD], 2680S; anti- β-tubulin III 1∶100, Sigma, T 8578) over 1 night at 4°C followed by incubation with the corresponding secondary antibodies (1∶200; Invitrogen, Carlsbad, CA) overnight at 4°C. The double-stained images were examined using an Axiovert/LSM 510 confocal scanning microscope (Carl Zeiss Microimaging, Inc., Germany). The experiment was repeated 3 times with similar results.

### Protein Extraction, Immunoprecipitation, and Western Blot Analysis

Spinal cord dorsal horn tissues from respective treatment groups of the L4-L5 ipsilateral sides were removed and immediately homogenized in ice-chilled lysis buffer (50 mM Tris, pH 7.4, 150 mM NaCL, 1.5 mM MgCL_2_, 10% glycerol, 1% Trition X-100, 5 mM EGTA, 0.5 µg/ml leupetin, 1 mM PMSF, 1 mM Na_3_VO_4_, 10 mM NAF, and a proteinase inhibitor cocktail). Homogenates were centrifuged at 12,000×g for 15 min at 4°C and protein concentrations determined using the BCA assay kit (Pierce, Rockland, IL). 30 µg of protein was separated by SDS-PAGE (sodium dodecyl sulfate polyacrylamide gel electrophoresis) and transferred on PVDF membranes. After blocking with 1% bovine serum albumin (BSA) in TBST (50 mM Tris-HCL, pH 7.5, 150 mM,NaCl, 0.05% Tween 20) for 1 h at room temperature, membranes were incubated overnight at 4°C with appropriate primary antibody. All antibodies were diluted in immuol staining primary antibody dilution buffer (CAN GET SIGANAL™, NKB-101, Toyobo, Japan) unless otherwise specified. Membranes were washed in TBST between all incubations. Blots were then incubated with a 1∶1000 dilution of horseradish peroxidase (HRP)-conjugated secondary antibody (goat anti-rabbit or -mouse, respectively) for 1.5 h at room temperature and respective bands visualized following incubation with enhanced chemiluminescence (Boehringer Mannheim, Indianapolis, IN) and exposure to x-ray file that were digitally analyzed using NIH Image software, version 1.60.

For immunoprecipitation, 50 µg of protein lysates from respective treatment groups of the L4-L5 ipsilateral sides were incubated with 2 µg of Cdk5 antibodies or with IgG as a control at 4°C for 1 h. Forty µl of protein G Sepharose (Amersham Biosciences) pre-washed with 1X PBS was added and mixed at 4°C for 1 h. After intense washing with lysis buffer 6 times, the immunoprecipitated protein and its associated proteins were analyzed by SDS-PAGE and Western blot analysis.

### Calpain Activity Assay

Calpain activity in protein lysates from respective treatment groups of the L4-L5 ipsilateral sides was analyzed using a commercially available calpain activity assay kit (BioVision, Milpitas, CA) following the manufacturer’s instructions. The kinase assay is based on fluorometric detection at an optical density of 505 nm that measures the cleavage of the calpain substrate Ac-LLY-AFC that emits blue light. Upon substrate cleavage, free AFC emits a yellow-green flurorescence, quantified using a fluorescence sensor (Tecan Spectra Fluor Plus, Vienna, VA).

### Statistical Analyses

All data were presented as mean ± SEM. Comparisons of differences between groups were analyzed using either the Student’s *t* test or by ANOVA. A Pearson’s correlation was used to establish correlations between groups. SPSS 12.0 for windows was used as the statistics software. The standard for statistical significance was p<0.05.

## Results

### Cdk5 Colocalizes with p35, Synaptophysin, and VAMP-2

**Figure 1:**
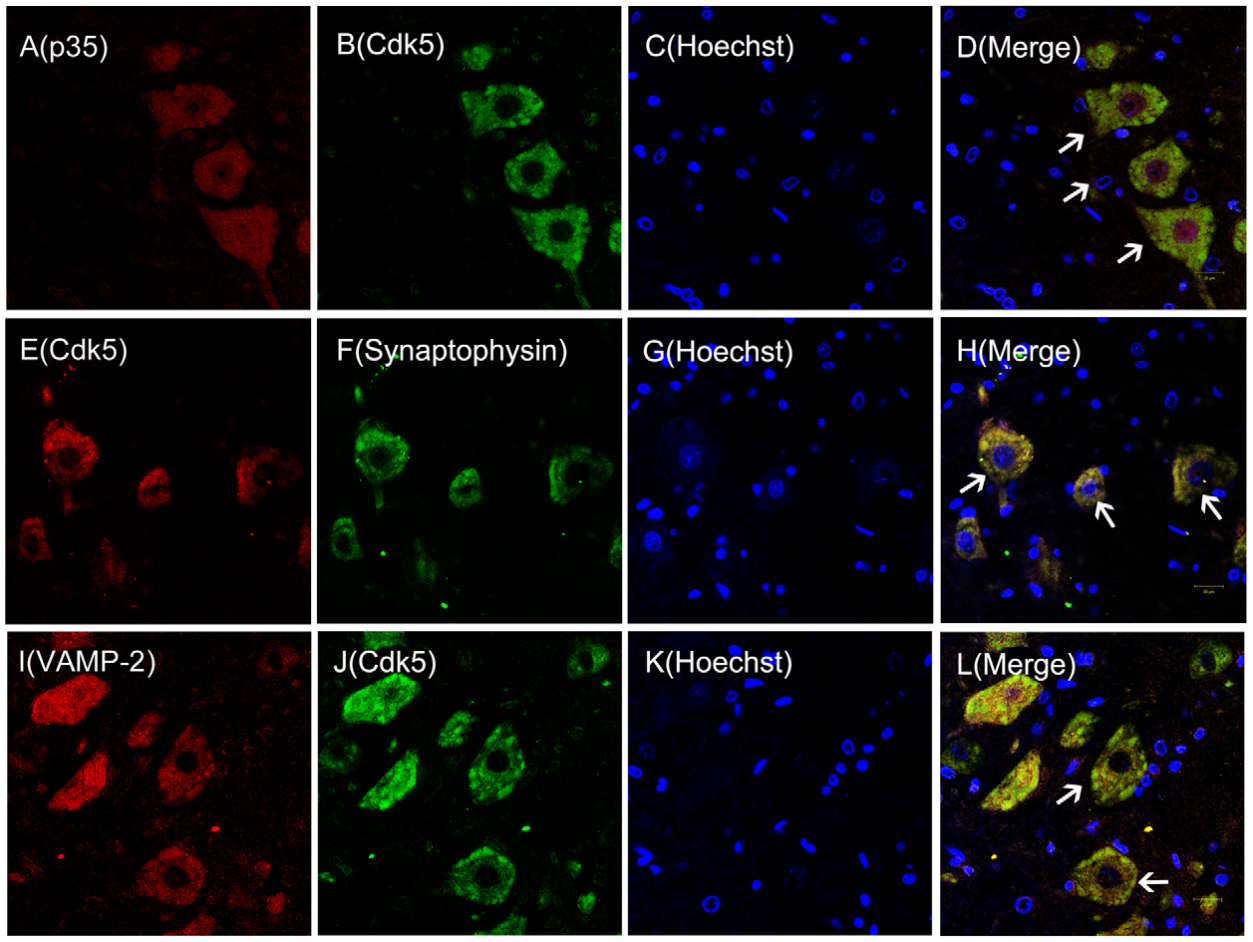
Cdk5 colocalizes with p35, synaptophysin, and VAMP-2. Double-immunofluorescence staining for p35 (red), Cdk5 (green or red), Hochest [a cell nuclear marker (blue)], synaptophysin (green) and VAMP2 (red) in ipsilateral spinal cord L4/L5 neuron sections of naïve rats (A–D). p35 colocalizes with Cdk5 (D, white arrow head).Cdk5 colocalizes with synaptophysin (H, white arrow head) (E–H). VAMP-2 colocalizes with Cdk5 (L, white arrow head) (I–L). (n = 3, per group, magnification X40, Bar = 20 µm).

Previous studies showed that presynaptic Cdk5 was distributed throughout the CNS [30]. It is well known that Cdk5 distribution and subcellular localization in tissues can help identify the functions of protein kinases and other proteins. Therefore, we determined whether Cdk5/p35 and synaptophysin/VAMP-2 colocalized (Figure 1). Double- immunofluorescence analysis of L4 and L5 spinal horn cord neurons indicated that Cdk5 colocalized with its activator p35 and synaptophysin. Furthermore, Cdk5 colocalized with VAMP-2 in the cytoplasm of neurons, indicating that Cdk5 may directly interact with synaptophysin/VAMP-2.

### Synaptophysin Colocalizes with SNAP-25, VAMP-2 and Syntaxin 1A

**Figure 2:**
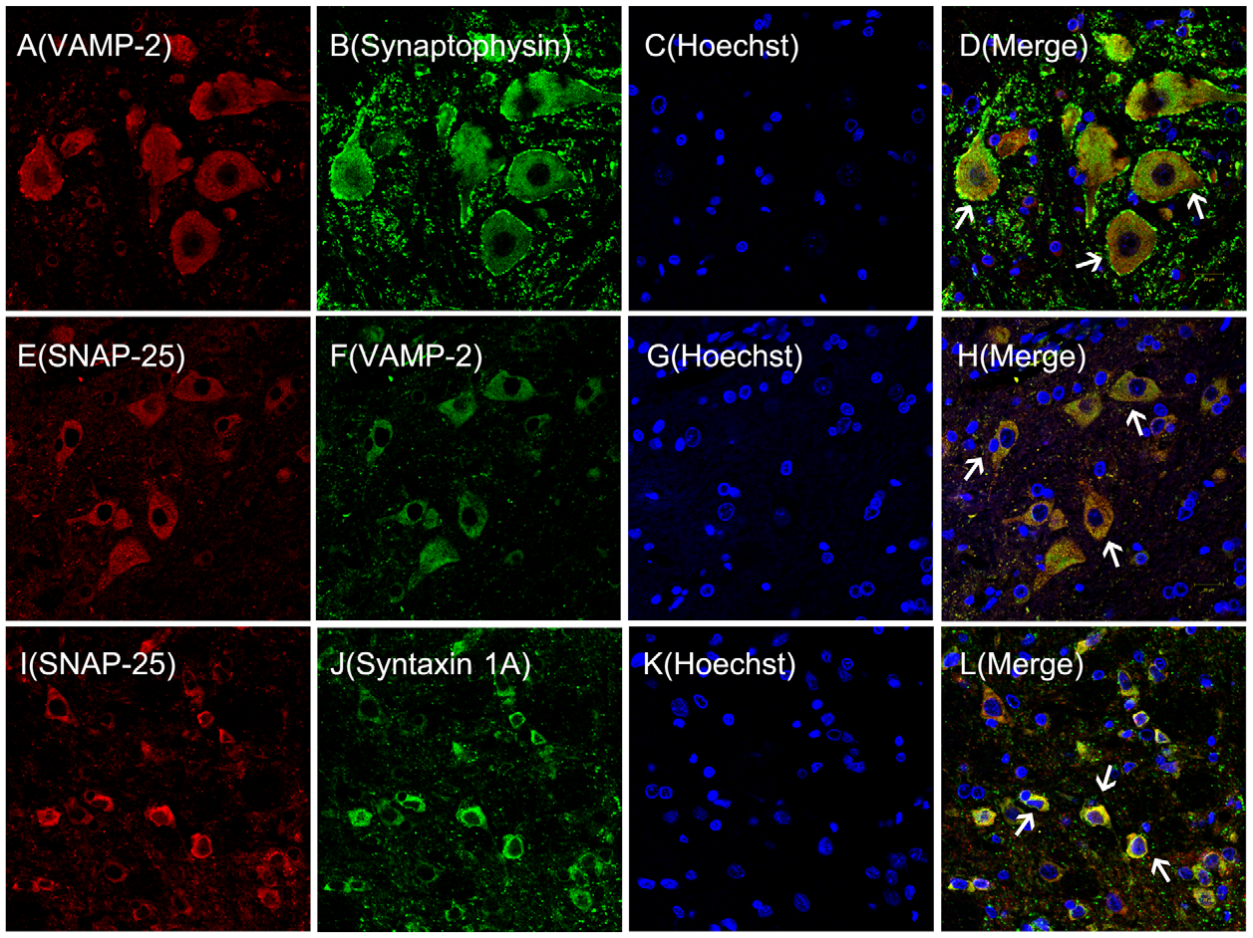
Synaptophysin colocalizes with VAMP-2, SNAP-25, and syntaxin-1A in spinal cord neurons. Double-immunofluorescence staining in the neurons of the ipsilateral spinal cord. L4 and L5 sections taken from naïve rats stained for VAMP2 (red), synaptophysin (green), Hochest,(blue), SNAP-25, and VAMP2 (red). VAMP-2 colocalizes with synaptophysin (D, white arrow head) (A–D). SANP-25 colocalizes with VAMP-2 (H, white arrow head) (E–H). SNAP-25 colocalizes with syntaxin-1A (L, white arrow head) (I–L). (n = 3/group, magnification X40, Bar = 20 µm).

Based on the close association between synaptophysin and SNAREs, we characterized their colocalization using double-immunofluorescence (Figure 2). This analysis demonstrated that synaptophysin colocalized with the key SNAREs components VAMP-2, SNAP-25, and syntaxin 1A in spinal horn cord neurons. Morphologically, the synaptophysin and Cdk5/p35 complex tightly colocalized with SNAREs in the same spinal horn cord neurons. The formation of Cdk5 and synaptophysin/SNAREs complexes were first reported in spinal cords, suggesting that Cdk5 may play a role in mediating inflammatory pain as a consequence of complex formation.

### Intrathecal Administration of Roscovitine Significantly Depressed Heat Hyperalgesia Induced by Intraplantar CFA Injection

**Figure 3:**
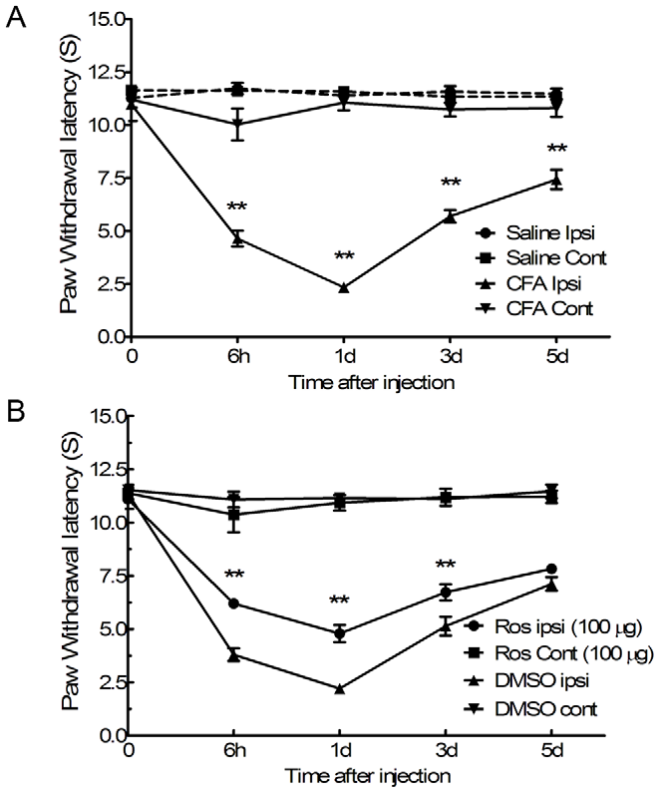
Heat hyperalgesia induced by intraplantar injection of CFA was inhibited by intrathecal administration of roscovitine. PWL to thermal stimuli significantly decreased after intraplantar injection of CFA; **p<0.01, n = 6, per group (A). PWL to thermal stimuli significantly increased following pre-intrathecal injection of roscovitine (Ros) 30 min before intraplantar injection of CFA compared to the PWL of the control group pretreated with DMSO, **p<0.01 (B). Intrathecal adminstration of roscovitine had no effect on the threshold of heat hyperalgesia of the contralateral paws of control groups. p>0.05, cont (contralateral) to hind paw injected with saline or CFA; Ipsi, ipsilateral to hind paw injected with CFA. Data are expressed as the mean ± SEM (n = 6/group).

It is well known that nociceptive behavior associated with inflammatory pain is linked to heat hyperalgesia. We observed the changes in nociceptive behavior following CFA injection by measuring thermal PWLs. In addition, we tested the effects of a selective Cdk5 inhibitor on the thermal PWLs. Following injection of CFA into rat ipsilateral hind paws, the peak of heat hyperalgesia presented 6 h post injection and lasted for 3 days. The average thermal PWL threshold (seconds) of ipsilateral hind paws was significantly reduced post CFA injection from 6 h to 5 d (6 h, 4.64±0.37 s; 1 d, 2.34±0.24 s; 3 d, 5.69±0.30 s and 5 d, 7.43±0.46 s) compared to contralateral hind paws in the same group during the same time span (6 h, 10.03±0.7 s 5; 1 d, 11.06±0.36 s; 3 d, 10.73±0.32 s and 5 d, 10.80±0.41 s, **p<0.01, Figure 3A, n = 6). However, differences in threshold of PWL between rat ipsilateral hind paws and contralateral hind paws challenged with saline remained unchanged in the control group (p>0.05, Figure 3A, n = 6). We found that the average ipsilateral paw PWL thresholds were remarkably increased following roscovitine pretreatment 30 min before intraplantar CFA injection between 6 h and 3 d post injection (6 h, 6.20±0.19 s; 1 d, 4.79±0.40 s; 3 d, 6.73±0.38 s) and remained unchanged on day 5 (7.83±0.19 s) post injection compared to ipsilateral paws from control group animals pretreated with DMSO during the 6 h to 3 d time period post injection (6 h, 3.80±0.30 s; 1 d, 2.21±0.24 s; 3 d, 5.14±0.44 s), and at 5 d post injection (7.12±0.32 s, **p<0.01, Figure 3B, n = 6). However, there were no differences between the thermal PWL of contralateral paws of rats pretreated with either roscovitine or DMSO, demonstrating that roscovitine had no effect on the heat hyperalgesia response of naive rats (p>0.05, Figure 3B, n = 6). In addition, the thermal PWL threshold of contralateral paws from rats pretreated with roscovitine did not change between 0 h and 5 d (0 h, 11.39±0.37 s; 6 h, 10.37±0.82 s; 1 d, 10.93±0.36 s; 3 d, 11.19±0.40 s; 5 d, 11.21±0.30 s) compared with the PWL threshold observed for contralateral paws of rats pretreated with DMSO (0 h, 11.53±0.25 s; 6 h, 11.08±0.37 s; 1 d, 11.16±0.22 s; 3 d, 11.11±0.16 s; 5 d, 11.47±0.3 s 2, p>0.05, Figure 3B, n = 6) demonstrating that roscovitine had no effect on the heat hyperalgesia response of naive rats.

### Synaptophysin Upregulation in Rat Spinal Cords following CFA Challenge was Reduced by Pretreatment with Roscovitine

**Figure 4:**
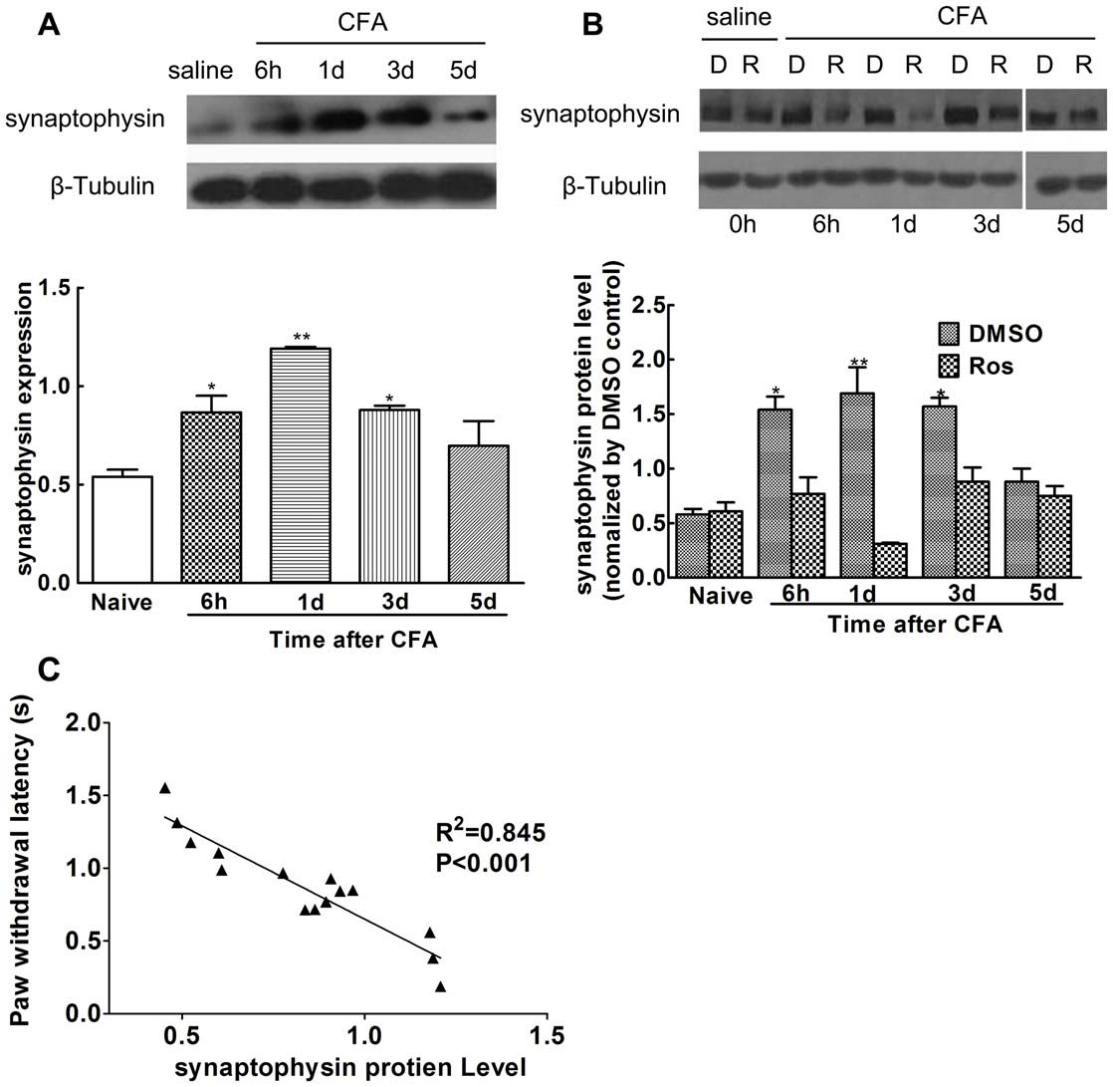
Synaptophysin levels were significantly increased in spinal cord dorsal horns and were inhibited by roscovitine. Compared to the control group, synaptophysin expression was significantly increased after intraplantar injection between 6 h and 3 d post CFA injection.(*p<0.05, **p<0.01) (A). Increased synaptophysin levels were decreased following intrathecal injection of roscovitine before CFA injection, compared to DMSO treated controls (*p<0.05, **p<0.01) (B). There were no significant differences between the roscovitine and DMSO treated groups treated with saline between 6 h and 3 d post CFA injection (p>0.05). Significant liner correlation was found between synaptophysin expression levels and the PWL thermal threshold (R^2^ = 0.845, p<0.001). Data are expressed as the mean ± SEM (n = 3/group).

Previous studies showed that heat hyperalgesia peaked 6 h post CFA injection and lasted over 24 h [31]. Therefore, synaptophysin protein expression in the L4/L5 ipsilateral spinal horn cords was analyzed by Western blot from 6 h to 5 d post CFA injection. Results demonstrated that synaptophysin expression significantly increased post intraplantar injection of CFA between 6 h and 3 d (6 h, 0.867±0.084; 1d, 1.191±0.009; 3 d, 0.879±0.084), and decreased (0.698±0.125) by 5 d compared to the control group (0.540±0.036, *p<0.05, **p<0.01, Figure 4A, n = 3). In addition, we found that administration of roscovitine decreased synaptophysin expression between 6 h and 3d (1.54±0.12 at 6 h; 1.69±0.24 at 1d, and 1.57±0.08 at 3 d compared to expression levels observed in the DMSO group (0.77±0.151 at 6 h; 0.31±0.01 at 1 d, and 0.88±0.13 at 3 d, *p<0.05, **p<0.01, Figure 4B, n = 3). However, intrathecal administration of roscovitine had no effect on synaptophysin expression in naive group rats or 5 d post CFA injection (i.e., levels in naive group animals were 0.58±0.05 versus 0.61±0.08, p>0.05, Figure 4B, n = 3 and on 5 d, 0.88±0.12 versus 0.75±0.09, p>0.05, Figure 4B, n = 3). Significant linear correlations were found between synaptophysin protein expression and PWLs (Figure 4C, R ^2^ = 0.845, p<0.001).

### Synaptophysin Significantly Interacted with Cdk5 Following CFA Challenge

**Figure 5:**
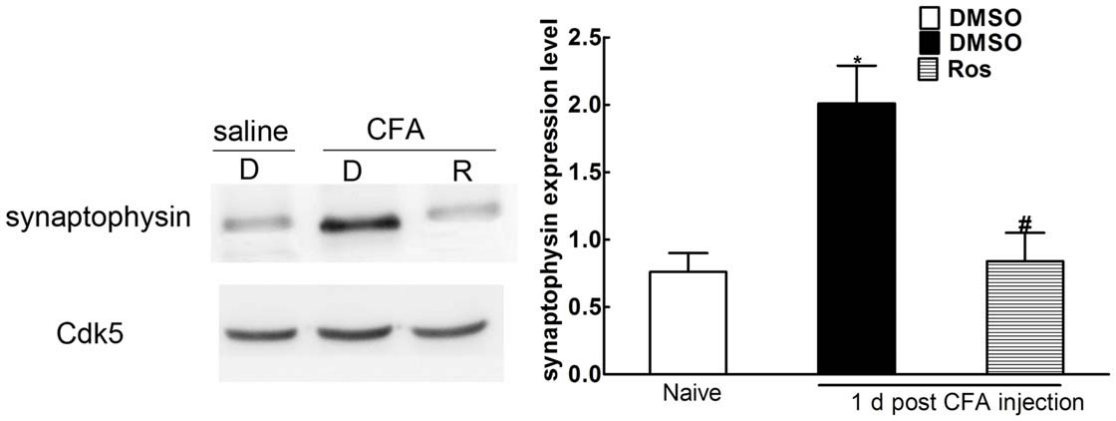
Synaptophysin closely interacted with Cdk5 in spinal cord dorsal horns post CFA injection. Compared to controls, synaptophysin (analyzed by Western blot after immunoprecipitation with Cdk5) was significantly increased. Increased synaptophysin levels were significantly inhibited by intrathecal injection of roscovitine 1 d post intraplantar injection of CFA (*p<0.05, ^#^p<0.05). Data are expressed as the mean ± SEM (n = 3/group).

Although significant upregulation of synaptophysin was inhibited by administration of roscovitine, it was insufficient to prevent the close interaction between Cdk5 and synaptophysin. Therefore synaptophysin protein expression in L4/L5 ipsilateral spinal horn cords was analyzed by Western blot following immunoprecipitation of Cdk5 and synaptophysin 1d post intraplantar injection of CFA. Compared to the control group, synaptophysin was significantly upregulated (0.76±0.14 versus 2.01±0.28, *p<0.05, Figure 5, n = 3). The increased synaptophysin levels were inhibited by intrathecal injection of roscovitine (2.01±0.28 versus 0.84±0.21, *p<0.05, Figure 5, n = 3).

### Calpain Activation was Transiently Upregulated Post CFA Injection

**Figure 6:**
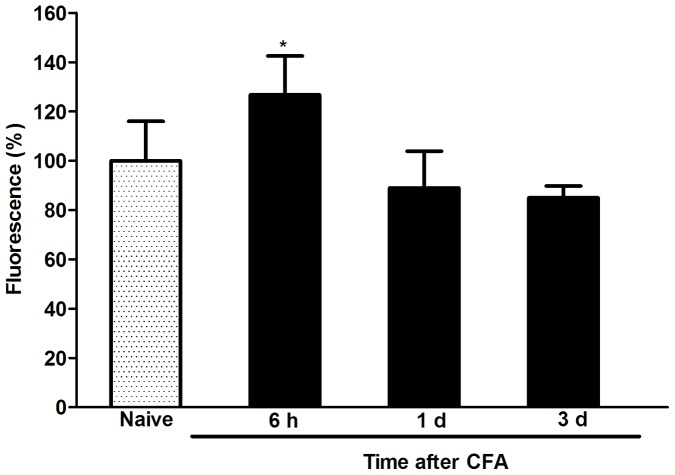
Calpain activity was transiently upregulated in spinal cord dorsal horns. Compared to controls, calpain activity was significantly upregulated 6 h (but not 1d and 3 d) post CFA injection (*p<0.05). Data are expressed as the mean ± SEM (n = 6/group).

Previous studies showed that calpain cleaved p35 into p25 [6]. Confirmation of calpain-mediated cleavage of p35 to p25 post CFA administration was assessed, demonstrating that calpain activity was significantly upregulated 6 h post CFA injection compared to calpain levels observed in controls (126.71±15.80 versus 99.99±16.01, *p<0.05, Figure 6, n = 3). However, no significant differences between 1 d (88.94±14.97) and 3 d (84.97±4.72) post CFA injection were observed compared to levels measured in the control group (99.99±16.01, p>0.05, Figure 5, n = 3).

## Discussion

This report demonstrated that synaptophysin was associated with heat hyperalgesia mediated by the presence of presynaptic Cdk5 in spinal cord dorsal horns of rats. Previous studies showed that Cdk5 and p35 were prominently expressed in spinal cord dorsal horn neurons of small or medium diameter which are sites where pain hypersensitization occurs [11]. Furthermore, results from previous studies indicated that Cdk5 mainly regulated the migration and apoptosis of neurons in the growth cone of immature neurons [31]. By contrast, presynaptic Cdk5 mainly mediated neurotransmitter release in the terminal axons of mature neurons [30]. However, this seems to depend on the context in which presynaptic Cdk5 is localized since it may either inhibit or promote neurotransmitter release [12]. The present study demonstrated that Cdk5/p35 colocalized with synaptophysin and SNARE (consisting of VAMP-2, SNAP-25, and syntaxin1A) in spinal cord dorsal horn neurons. It is well known that the presynapse is the structure where synaptic vesicles containing-neurotransmitters fuse to the synaptic membrane and are recycled into internal compartments by exocytosis and endocytosis, respectively [20]. Our results demonstrated that colocalization of Cdk5 and synaptophysin/SNAREs in the presynapse may suggest that Cdk5 mediated the excitatory neurotransmitter release and transfer of nociceptive messages by acting on synaptophysin in these tissues.

Results from previous studies showed that peak heat hyperalgesia responses induced by peripheral CFA injection began at 6 h and lasted at least 24 h post injection [32]. Our results indicated that synaptophsin levels increased between 6 h and 3 d during maximum heat hyperalgesia induced by intraplantar injection of CFA. In addition, there was a significant correlation between synaptophysin expression levels and the thresholds of thermal PWL. In other models, Cdk5 knockout led to a decrease of synaptophysin expression in the forebrains of mice [22]. In addition, the results from a previous report demonstrated that the reduced synaptophysin resulted in the compensatory increase of Cdk5 in the CA1 of hippocampus in mice [33].In our model, intrathecal adminstration of roscovitine significantly inhibited synaptophysin expression 6 h post CFA injection up to day 3. Immunoprecipitation was then used to confirm the interaction between Cdk5 and synaptophysin in the spinal cord dorsal horns of CFA-treated rats. Immunoprecipitation of Cdk5 and synaptophysin indicated that synaptophysin was significantly upregulated 1 d post intraplantar injection of CFA, and increases to the synaptophysin levels were significantly inhibited by intrathecal injection of roscovitine suggesting that there was a significant interaction between Cdk5 and synaptophysin post intraplantar injection of CFA, and that Cdk5 mediated the heat hyperalgesia induced by peripheral injection of CFA via acting on synaptophysin.

Using this same model, others demonstrated that Cdk5 activity was significantly upregulated from 2 h to 7 d in the spinal cords of CFA-treated rats. Their results indicated that p25 (but not p35) was upregulated from 6 h up to 7 d post peripheral injection of CFA [11]. It was previously shown that calpain cleaved p35 (into p25) following Cdk5 activation [6]. In our study, calpain activity was only upregulated post 6 h CFA administration. However, since both p25 levels and calpain activity were upregulated in the DRG and spinal cords of mice challenged with carrageen in the absence of decreased p35 levels, it suggested that p25 was not generated as a result of calpain-mediated cleavage of p35. Furthermore, determining the mechanism associated with changing p25 levels cannot be carried out using calpain inhibitors [9]. No significant changes in p35 activity were observed in the spinal cord dorsal horn neurons although both p25 and Cdk5 activity was significantly upregulated between 6 h up to 5d post peripheral injection of CFA [11].

Previous studies showed that calpain was upregulated in the spinal cords of rats post plantar injection of zymoson [34]. In addition, the calpain III inhibitor MdL-28170 moderately reversed the decreased thresholds of thermal PWL induced by plantar injection of zymoson. However, a different calpain inhibitor class E-64d significantly reversed the decreased thresholds of thermal PWL caused by nerve injury, but not by plantar injection of CFA in mice [35]. In the model described in this study, increased calpain activity was only significantly upregulated in spinal cords 1 d post CFA injection. The differences between results obtained in our study and results reported by others may have been due to differences between animal models used.

In addition, other studies showed that the activity of p25 isolated from the brains of Alzheimer's patients (derived from cleavage of p35) was partly dependent on activated calpain [36]. Previous results obtained by other laboratories indicated that p25 was significantly upregulated from 2 h to 7 d post plantar injection of CFA in rats [11]. Based on the calpain data presented in this report, we suggest that p25 was generated via a p35-independent pathway and that calpain did not play a critical role in activating Cdk5 kinase activity in the CFA-treated inflammatory pain model.

Considerable evidence has demonstrated that SNARE formation was prerequisite to neurotransmitter release [37]. During the course of SNARE formation, VAMP-2 docks with SNAP-25/syntaxin 1A to make synaptic vesicles fuse with the synaptic plasma [38]. Meanwhile, the movement of VAMP-2 toward the synaptic plasma membrane is the first stage of SNARE formation [39]. Fusion of synaptic vesicles with the synaptic plasma membrane is a deciding factor for synaptic vesicles mediating neurotransmitter release [40]. However, our study showed that synaptophysin colocalized with VAMP-2 in spinal cord neurons based on the observation that the migration of VAMP-2 required the participation of synaptophysin [40], [41]. These *in vitro* and *in vivo* data demonstrated that synaptophysin played various roles associated with endocytosis and exocytosis of synaptic vesicles, and that synaptophysin also played a leading role in SV trafficking [17]. The present study indicated that expression of synaptophysin was significantly upregulated in the spinal cord dorsal horns from 6 h to 3 d post intraplantar injection of CFA. During this time frame, the release of glutamate mediated by synaptophysin may have played a key role in mediating the heat hyperalgesia induced by peripheral injection of CFA.

The course of continuous neurotransmitter release resulting from endocytosis and exocytosis events was facilitated by synapse vesicle proteins that transport synapse vesicles. Additionally, a complex series of protein-protein interactions was associated with the synapse vesicle cycle [42]. Previous results showed that synaptophysin controls the targeting of VAMP2 to synaptic vesicles to mediate neurotransmitter activity [43]. Following a Ca^2+^ influx into the synaptic plasma membrane, synapse vesicles fused with the synaptic plasma membrane to release neurotransmitters. Therefore, the binding of synaptophysin to VAMP-2 becomes the starting point initiating neurotransmitter release [44], suggesting that the formation of the SNARE complex containing both synaptophysin and VAMP-2 was the hallmark of synapse maturation [45].

Many synaptophysin functions associated with the control of synapse vesicle recycling remain controversial [46], [47]. However, emerging evidence showed that synaptophysin was specifically required for endocytosis of synaptic vesicles [48]. Additional experiments utilizing imaging and electrophysiological analyses demonstrated that synaptophysin was indispensable for efficient endocytosis of synaptic vesicles in cultured hippocampal neurons [49].

Previous studies have also demonstrated the essential role played by Cdk5 during synaptic vesicle endocytosis and trafficking of SV [50], [51]. Single and double synaptophysin (or of the homologous protein synaptogrin) knockouts severely decreased short- and long-term synaptic plasticity in the CA1 region of the hippocampal [52]. Increasing experimental evidence has further shown that the formation and increase of synaptic plasticity in CNS played a critical role in mediating chronic neuropathic and inflammatory pain [53], [54], [55]. In our model, the level of synaptophysin expression significantly correlated with the duration from 6 h to 3 d post peripheral injection of CFA, which might suggest that synaptophysin was involved in inducing and maintaining long-term synaptic plasticity associated with central pain sensitization. Blocking Cdk5 function resulted in a significant decrease in the amount of synaptophysin clusters present in mouse brain, demonstrating that Cdk5 was vital to increasing synaptic plasticity [22].

Although roscovitine significantly inhibited Cdc2, Cdk2, Cdk5, and Erk1/2 kinase activity [56], roscovitine mainly inhibited Cdk5 activity associated with synaptophysin for the following reasons: 1) Cdk5 was primarily confined to the postmitotic neurons, and Cdc2 and Cdk2 were expressed only at the embryonic stage [57], [58]. 2) All Cdk kinase activities were silenced in mature neurons except Cdk5 [59], [60], [61]. 3) The results from previous experiments demonstrated that the action of roscovitine in mediating neurotransmitter release in the hippocampal CA3-CA1 region of rats was mediated by presynaptic Cdk5 rather than via an independent Cdk5 pathway [12]. 4) In our study, we used adult rats to test the effects of roscovitine on synaptophysin present in spinal cord dorsal horn neurons. 5) Even though roscovitine was capable of inhibiting Erk1/2 functions associated with mediating pain signaling via the spinal cord, previous studies showed that roscovitine had no effect on activated p-ERK1/2 in spinal cord dorsal horns post peripheral injection of CFA [11]. 6) Furthermore, immunoprecipitation of Cdk5 and synaptophysin demonstrated that the significant upregulation and downregulation of synaptophysin was closely associated with Cdk5. Taken together, increased synaptophysin levels were involved in heat hyperalgesia mediated by Cdk5 in the spinal cord dorsal horns of rats that were treated with CFA. However, future experiments will be designed to further define the interactions between Cdk5 and synaptophysin.

The interaction between synaptophysin and Cdk5 in the context of mediating heat hyperalgesia induced by peripheral injection of CFA has not been previously reported. We speculated that the role of synaptophysin in mediating heat hyperalgesia via Cdk5 may promote or prevent SNARE formation by increasing or decreasing synaptophsin concentrations. On the other hand, previous studies showed that synaptophsin promoted the release of the neurotransmitter by binding Ca^2+^ to the cytoplasmic domain of the transmembrane polypeptide [62], [63]. Therefore, roscovitine may mediate Ca^2+^ influx into presynaptic vesicles by mediating P/Q type voltage-dependent calcium channels (VDCC) in our model. The glutamatergic neuron system plays a key role in mediating inflammatory pain [64]. Increasing evidence has showed that Cdk5 presynaptically functioned in controlling neurotransmitter release, *e.g*. glutamate [13]. However, even though other studies have showed that synaptophysin mediated the release of glutamate in other models [62], we wondered if and how Cdk5/synaptophysin mediated the release of glutamate in our model. Since we did not test the interactions between synaptophysin and SNAREs, future experiments will have to be carried out to gain a better understanding of these interactions. Nevertheless, data presented in this study have opened the door for future experiments designed to study Cdk5-mediated pathways associated with inflammatory pain via presynaptic mechanisms.

### Conclusions

Data presented in this study demonstrated that the increased levels of synaptophysin in spinal cord neurons resulting from the peripheral injection of CFA were significantly associated with nociceptive responses. Intrathecal administration of roscovitine significantly inhibited synaptophysin expression, which may be important in blocking the initiation or early phases of chronic inflammatory pain. Therefore, preventing activation resulting from Cdk5/synaptophysin interactions may represent a novel therapeutic anti-inflammatory pain target.
